# Chemotherapy use and quality of life in cancer patients at the end of life: an integrative review

**DOI:** 10.1186/s12955-020-01580-0

**Published:** 2020-10-07

**Authors:** Elham Akhlaghi, Rebecca H. Lehto, Mohsen Torabikhah, Hamid Sharif Nia, Ahmad Taheri, Ehsan Zaboli, Ameneh Yaghoobzadeh

**Affiliations:** 1 School of Nursing and Midwifery, Iran University of Medical Sciences, Tehran, Iran; 2 College of Nursing, Michigan State University, East Lansing, MI USA; 3 Department of Adult Health Nursing, Nursing and Midwifery Faculty, Isfahan University of Medical Sciences, Isfahan, Iran; 4 Department of Nursing, Mazandaran University of Medical Science, Sari, Iran; 5 Gastrointestinal Cancer Research Center, Mazandaran University of Medical Sciences, 48166-33131 Sari, Iran; 6 Tehran University of Medical Sciences, Tehran, Iran

**Keywords:** Quality of life (QOL), Health related quality of life (HRQOL), Cancer chemotherapy, Drug therapy, End of life care, Palliative care, Palliative therapy, Palliative treatment

## Abstract

**Background:**

When curative treatments are no longer available for cancer patients, the aim of treatment is palliative. The emphasis of palliative care is on optimizing quality of life and provided support for patients nearing end of life. However, chemotherapy is often offered as a palliative therapy for patients with advanced cancer nearing death. The purpose of this review was to evaluate the state of the science relative to use of palliative chemotherapy and maintenance of quality of life in patients with advanced cancer who were at end of life.

**Materials and methods:**

Published research from January 2010 to December 2019 was reviewed using PRISMA guidelines using PubMed, Proquest, ISI web of science, Science Direct, and Scopus databases. MeSH keywords including quality of life, health related quality of life, cancer chemotherapy, drug therapy, end of life care, palliative care, palliative therapy, and palliative treatment.

**Findings:**

13 studies were evaluated based on inclusion criteria. Most of these studies identified that reduced quality of life was associated with receipt of palliative chemotherapy in patients with advanced cancer at the end of life.

**Conclusion:**

Studies have primarily been conducted in European and American countries. Cultural background of patients may impact quality of life at end of life. More research is needed in developing countries including Mideastern and Asian countries.

## Introduction

Best practices for management of advanced cancer are of global health concern, particularly in developing countries [1]. When curative strategies are exhausted, the focus of cancer care shifts to maintenance of quality of life (QOL) and extension of survival [2]. Studies indicate that – 20 to 50% of patients with advanced cancers receive chemotherapy (CT) towards end of life with the aims of extending survival and improving QOL [3–5]. Such rigorous treatment strategies may contribute to death in non-preferred environments such as the intensive care unit (ICU), incurring additional costs for care that is often futile and that may also diminish QOL in the patients’ final days. Intensive medical management at end of life may also limit the opportunity for patients to receive hospice services, supportive care that focuses on enhancing comfort and promoting quality of life when facing death [6]. The purpose of the review was to examine the state of the science relative to the use of CT in patients with advanced cancers at the end of life, and to evaluate the impact of such treatments on patients QOL as they near death.

Chemotherapy generally refers to treatments aimed at stopping or eradicating the growth of cancer cells that are administered orally, intravenously, intrathecally, by injection, or subcutaneously depending on the type and stage of cancer being treated [7]. For patients with advanced cancers, many are now receiving oral antineoplastic agents [8]. Chemotherapies (CT) in general are associated with a host of side effects and symptoms that are specific to the type of agent and individualized patient characteristics. Numerous studies have identified the impact of CT on QOL, with some research suggesting that patients may experience improved QOL and length of survival when offered such treatments as a last alternative [9]. However, CT are also associated with increasing the cost of care at end of life due to the additional costs associated with need for pharmaceuticals, potential for blood products, laboratory and diagnostic testing, health professional support and unscheduled visits that result in hospitalization [10, 11].

Palliative care is aimed at providing comprehensive support and comfort via a multidisciplinary team approach to enhance QOL when cure is not an option [12]. Some research has shown that stoppage of intensive medical treatments and providing palliative care have improved both QOL and length of survival of advanced cancer patients [13]. Further, the incorporation of early specialized palliative care may reduce the risk of receiving CT during the final two weeks of life [14]. Many patients who have good performance status, however, receive palliative CT as they near end of life with the aim of increasing survival length and even to improve QOL [10].

According to the Health Service Research Committee of the American Society of Clinical Oncology (ASCO), CT can potentially improve QOL in late stages of life even if it doesn’t impact survival length [13]. Further, medical decision making relative to use of CT in patients nearing the end of life may be fostered by the development and availability of newer anticancer agents that have fewer side effects [15]. However, ASCO and the National Quality Forum have identified that other factors impact QOL at end of life (defined as final 30 days of life) and should be considered, such as unscheduled emergency room visits, the potential for lengthy hospitalizations, ICU admissions, and ICU deaths [16]. Recently, ASCO contended that stopping CT in cancer patients at the end of life is one of five factors which can improve the quality of patient care and reduce healthcare costs [16]. There remain limited studies that have systematically evaluated the effects of palliative CT on the QOL of cancer patients nearing end of life [4, 16, 17]. Some studies evaluate factors such as age, sex, type of cancer, and health care system characteristics while omitting information about perceived QOL [14]. Research findings suggest that culture may impact the utilization of aggressive treatment in patients with advanced cancer. For example, a study from Japan stated that only 3.7% of patients receive CT in their last 2 weeks of life [18]. In spite of increasing use of CT at end of life in recent years, the impact on patients' QOL remains equivocal. Therefore, the purpose of this review was to evaluate what is known about the use of CT and associated QOL in patients with advanced cancer approaching end of life.

## Methods

### Research question

The research question aimed to examine: what is the state of the science between use of palliative CT and QOL in patients with advanced cancer at end of life?

### Search strategy

**Fig. 1:**
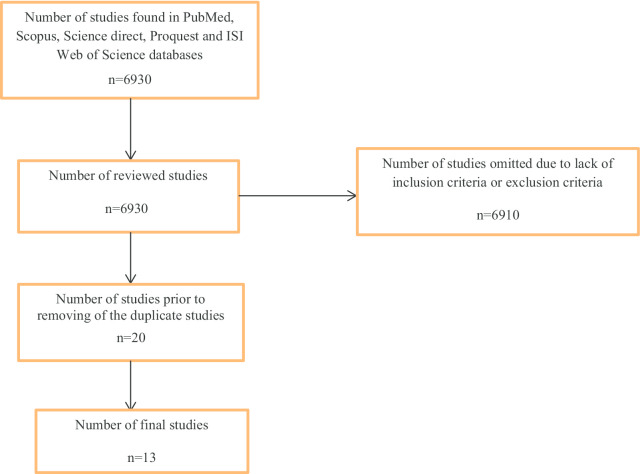
Systematic review search based on PRISMA

An integrative review was conducted in 2019. Studies published between January 2010 and December 2019 were reviewed using PRISMA guidelines (see Fig. 1). Six databases (PubMed, Proquest, ISI web of science, Science Direct, Scopus, SID) were searched using AND and OR Boolean operators. MeSH keywords including quality of life (QOL), health related quality of life (HRQOL), cancer chemotherapy, drug therapy, end of life care, palliative care, palliative therapy, and palliative treatment were used to search for associated studies.

### Eligibility criteria

Inclusion criteria were: (1) Persian and English articles published between January 2010 to December 2019 that were freely accessible; (2) patients with advanced cancer at the end of life; (3) evaluation of anti-cancer CT and patients' QOL; and (4) quantitative data based (including retrospective, prospective cohort, cross-sectional studies,). Exclusion criteria were studies that evaluated specific CT regimens without an inquiry of patients' QOL, and use of other treatment modalities in conjunction with CT. Also eliminated were interventional studies that did not address the relationships between use of palliative CT and QOL in patients with advanced cancer nearing end of life. The Boolean operators like OR (for synonymous keywords) and AND (for relating different keywords) was performed to search the articles. Utilizing these established inclusion and exclusion criteria, abstracts of retrieved articles were first reviewed and those that matched the criteria were evaluated. After duplicates were removed, two researchers independently reviewed the included articles and concurrence was achieved with 13 papers finally selected. The search results are presented in Table 1. The steps for choosing articles based on PRISMA guidelines are also shown in Fig. 1.

**Table 1:** Number of articles found from respective databases

Databases	Number of articles	Selected articles
PubMed	5627	7
Proquest	960	6
ISI web of Science	166	5
Science Direct	90	0
Scopus	87	2
SID	0	0
Total	6930	20

### Quality assessment (risk of bias)

The quality of the studies was separately measured by two researchers using two tools, the Quality Assessment of Diagnostic Accuracy Studies (QUADAS-2) and the checklist of Standards for Reporting of Diagnostic Accuracy (STARD) [19]. These tools examine the risk bias in accuracy and comprehensiveness of study reports. The combination of both tools, with revision of some cases, improved the examination of the included studies [20]. QUADAS-2 was used to measure the internal stability of the studies studied. The tool consists of items with three-option answers (i.e., yes, no, and unknown). A 25-item STARD tool was used to evaluate the study design quality (including sample collection and data). The combination of these two tools and their associated flexibility helped make them compatible with the study goals [20]. Standardized methods were used to ensure the quality assessment and to ensure the inter-rater reliability of both analyses. Finally, findings from the two assessments were evaluated to ensure that they were comparable [21].

## Results

Of the 13 studies, five studies were conducted in the United States (U.S.), six were from Europe, and one were from Australia and India respectively. Based on studies that reported this information, the sampling interval across the 13 studies were from 2002 to 2012 (Table 2).

**Table 2:** Summaries of the information and results of the reviewed studies

Author/s (year)	Country	General sample characteristics	Type of study	Sampling interval	Results
Bisht et al. (2011)	India	40 head and neck cancer patients; Types included oral (n = 12), laryngeal (n = 12), pharyngeal (n = 8), nasal sinus (n = 3), salivary gland (3 patients), thyroid (n = 2)	Prospective, observational cohort study	Not indicated	Treating symptoms such as pain aggressively can improve the QOL of patients with advanced stage head and neck cancers. None of the patients died during the 2 month study
Fujisawa, et al. (2015)	U.S	Out of 125 patients with non-small cell lung cancer, 25 (20%) who received CT in final 14 days of life; 11 females; 14 males; mean age 64 years; 15 received oral CT, 10 received IV CT	Secondary analysis of data from randomized control trial	2006–2009	Patients who received CT at end of life had significantly higher anxiety, depression, and poor psychological QOL
Groene.et al. (2015)	England	2313 patients who received palliative CT for advanced esophageal/gastric cancers; 27% male. 51% of sample under age 55 years; 9% over age 75 years	Prospective population-based observational study	2007–2009	Completion of palliative CT is low, particularly in the elderly with low functional status. The study had high levels of missing data, but findings concluded the need to carefully weigh the treatment-risk benefit ratio in terms of toxicities and QOL
Hui et al. (2014)	U.S	Evaluated 113 (14%) patients with hematologic malignancies with rest of 816 patients who died of advanced cancer who were under care at major cancer center. 52% male, Mean age 62 years, 61% white	Retrospective cohort	2009–2010	Patients with hematologic malignancies had poorer QOL at death including receipt of more aggressive care in final 30 days of life
Kao et al. (2013)	Australia	147 patients with malignant pleural mesothelioma, 12% of whom received chemotherapy in last month of life	Retrospective review	2007–2009	Those who received end-of-life CT had a significantly shorter survival time. Palliative CT did not improve QOL
Mayrbäurl et al. (2016)	Austria	100 advanced colorectal cancer patients; 60% men; mean age 66.4 years	Prospective observational study	2007–2011	25%, 29%, and 26% of patients died in the 1st, 2nd, and 3rd year of the study. The mean survival time was 21.8 months. QOL deteriorated over time, in particular among those receiving third line palliative CT
Mayrbäurl et al. (2012)	Austria	104 patients with advanced cancer Major types included colorectal (30.4%), Breast (11.8%), head/ neck (10.8%); and lung (9.8%); male (56.7%); mean age 66.2 (10.7)	Prospective observational study (included a healthy age and sex-matched control comparison group)	2003–2005	Patients had substantially lower QOL as compared to healthy controls. Patients receiving third-line or more palliative CT had worse QOL. The average survival rate of patients was 17.5 months. Patients who received first-line CT had a higher survival rate at the end of the study as compared with those receiving more CT
Näppä et al. (2011)	Sweden	374 patients with various types of incurable cancers (87 received palliative CT)	Cohort	2007–2008	Patients receiving CT in final month of life were less likely to die in preferred environments, had shorter survival, and had more frequent hospital admissions
Pacetti et al. (2015)	Italy	Out of 1001 cases with advanced cancers, 324 (24%) received palliative CT in final 30 days. Mean age 67.8 years; 64% males. Italian sample	Retrospective cohort	2010–2012	The patients who died during ongoing CT were being treated in 3^rd^-5^th^ lines of therapy. To improve patients QOL, concurrent care is needed to reduce usage of CT at end of life
Prigerson et al. (2015)	U.S	384 patients who died; 158 of whom received CT. Sample 55% male; mean age 58.6 years, 61.5% white; 20.5% black; 16.7% Hispanic	Cohort	2002–2008	Palliative CT in late stages of life QOL at death, especially in people with better functional status
Wright et al. (2014)	U.S	386 adult patients with metastatic cancer	Prospective cohort	2002–2008	Patients who underwent palliative CT were more likely to receive CPR, mechanical ventilation, and feeding tubes in the last week of life. These patients were more likely to die in intensive care than at home. Palliative CT was associated with factors that lower QOL at end of life
Wintner et al. (2013)	Austria	263 outpatients with advanced lung cancer	Prospective observational study	Not indicated	QOL was decreased in patients receiving more CT. These patients had disease progression and more treatment side effects
Zhang et al. (2012)	U.S	396 patients (mean age 58.7 ± 12.5 years; 65% white), with various types of end stage cancers	Prospective cohort	2002–2008	Dying in the hospital and/or in the Intensive care unit associated with lower QOL at death. Limiting use of CT and feeding tubes and transferring patients to hospice or to home may improve QOL at death

### General characteristics of the included studies

The included studies incorporated patients with advanced cancers who had undergone palliative CT. Sample sizes ranged from 40 [22] to 2313 [23] participants with an age range between 50 and 70 years old. Most study participants were male (see Table 3 for demographic characteristics). Quality of life was measured primarily with questionnaires including the Quality of Life Questionnaire-C30 (EORTC QLQ-C30), an internationally validated reliable instrument that evaluates QOL in social, role, cognitive, emotional, and physical functioning domains [16, 18, 24]. The European Cooperative Oncology Group (ECOG) performance status [3, 4, 16], the McGill Quality of Life Questionnaire [25], and Padilla’s Quality of Life Index [14] were also used. Other factors use to evaluate QOL included place of death and use of aggressive therapies at end of life [3, 12].

**Table 3:** Demographic characteristics of the studied units in the selected studies

Sex (male)		Age		Number of chemotherapy samples	Total number of samples	Study
The number of recipients of palliative chemotherapy (male)	Total Number (male)	Medium age recipients of palliative chemotherapy	Average age			
–^a^	28	–^a^	55	24	40	Bisht
63	–^a^	64.5	64	125	151	Fujisawa
1717	6339	55	–^a^	2313	9768	Groene
–^a^	182	–^a^	62	350	816	Hui
7	135	–^a^	73	9	147	Kao
60	60	66.4	66.4	100	100	Mayrbäurl 2016
59	59	66.2	66.2	104	104	Mayrbäurl 2011
49	190	65	65.5	87	374	Napa
105	105	67.8	67.8	162	162	Pacetti
85	171	56.3	58.6	158	312	Prigerson
119	215	56.4	58.4	216	386	Wright
112	112	67.1	67.1	187	187	Wintner
216	389	–^a^	–^a^	396	396	Zhang

^a^Information not available

### Main outcomes

Prigerson et al. (2015) conducted a prospective, multi-institution cohort study of patients with end-stage solid tumor cancers from different cancer centers across the U.S. to determine the relationships between use of CT and QOL as patients neared death. Out of 660 patients with late stage cancer, 384 died during the study period and these patients were evaluated as they approached death. Of these patients, 158 (about half, were receiving CT at study enrollment (4 months median time before death). The patients who received CT were significantly younger, better educated, had few comorbid conditions, had better performance status and were more likely to receive treatment at academic cancer centers. Further, they were more likely to have either breast or pancreatic cancer diagnoses. Findings demonstrated that patients with better performance status had a worse quality of death even when controlling for intensive care at end of life. For those patients with low to moderate performance status, CT use was unrelated to quality of death. The researcher’s findings indicated that CT use at end of life had no benefits to length of survival, and impaired QOL in the patients’ final days [4].

A secondary analysis of data from a randomized clinical trial evaluating palliative care for patients with advanced non-small cell lung cancer were analyzed to determine predictors of use of CT in the final two weeks of life. Study findings demonstrated that of 125 patients who died, 20% (n = 25) had received CT at end of life. Of these 25 patients, 15 were receiving oral CT whereas 10 were receiving intravenous CT or a combination of both [25]. Patients who received CT at end of life had worse psychological health including significantly more anxiety, depressive symptoms, and poorer QOL [25].

Hui and colleagues conducted a retrospective cohort study of patients with advanced cancer who died while receiving care at a major U.S. Cancer Center. They conducted a comparative analysis of care for 113 patients with hematologic malignancies (14% of sample) with patients who had solid tumor cancers (n = 816) [16]. The patients who died with hematologic malignancies were significantly more likely to receive aggressive treatments in their final 30 days compared to the group of patients with solid tumor cancers. Such care included ICU admissions, CT and targeted therapies, prolonged hospitalizations, and emergency unscheduled use of services [16] that culminated in lower QOL at death.

In a retrospective cohort study evaluating patients with advanced metastatic cancer during their last month of life, Pacetti et al. (2015) found that 162 patients out of 2164 at a major Italian Oncology Center received CT prior to death. Of these patients who received CT in their final 30 days, about 65% were males, and the individual health provider was the only predictor for why CT was continued at end of life [13].

Wright et al. (2014) conducted a secondary analysis of data from a large federally funded prospective, longitudinal, multi-institutional study in the U.S. of terminally ill patients with advanced cancer. Data from 386 patients who died during the study were analyzed to determine whether palliative CT administration in the final months of life was related to intensity of medical care and location of death [3]. Patients who received palliative CT (56% of the sample) as compared with those who did not receive palliative CT were significantly more likely to die in a non-preferred environments, were more likely to be hospitalized in an ICU at time of death, and were also more likely to receive cardiopulmonary resuscitation and/or ventilator support [3].

In a retrospective review study, Kao et al. (2013) evaluated factors associated with use of CT at the end of life in Australian patients with malignant mesothelioma. Of 147 patients who died, 21 received treatment in the last month of life [26]. For those patients receiving CT during the last month of life, the only factor associated with its’ use was having at least two previous cycles of treatment. These patients who received CT had significantly shorter survival as compared to patients who did not receive CT and there was a trend towards death outside the home. A limitation of the study was a lack of information about where these deaths occurred. Importantly the study suggests that end of life planning is less likely to occur when patients continue with CT in the terminal phase of life [26].

In a study from Sweden, Nappa and his colleagues (2011) evaluated characteristics associated with use of palliative CT in patients with advanced stage epithelial cancers in the last month of life. Of 374 patients, 87 (23%) received palliative CT in the last month of life [24]. Use of palliative CT was associated with shorter survival, increased hospital admissions, and less likelihood of dying at home. Further, there was less documented evidence of discussion about ceasing treatment with patients who received palliative CT during the last month of life.

In a large federally funded longitudinal prospective cohort study in the U.S., factors associated with QOL at end of life were evaluated in 396 patients with advanced cancer and their caregivers. Factors that were most associated with lower QOL at end of life included death in intensive care and receipt of life-prolonging therapies [2]. Better patient QOL was associated with positive relationships with their physician and pastoral spiritual care provision [2]. Other factors that improved QOL at death included personal religious activities, caregiver health, and better perceived mental health [2].

In another prospective cohort study, Bisht et al. (2011) evaluated 40 patients with advanced stage head and neck cancer who were receiving palliative care. All patients had stage 4 cancer and had recurrence and metastasis to distant areas. Of the 40 patients, 24 received palliative CT, in conjunction with other medications such as analgesics, steroids, and anti-emetics for symptom management. Findings identified that QOL was positively impacted by pharmacologic management of symptoms [22].

Mayrbaurl et al. (2016) conducted a prospective observational study that evaluated the QOL of 100 patients with advanced colorectal cancer who received first, second, or third lines of palliative chemotherapy and were followed for up to 3 years [27]. At the onset of the study, 73 patients began first-line of palliative CT, and 27 were at the start of second-line palliative CT. Ongoing QOL assessment was continued until patients’ were unable to complete the questionnaires, death occurred, or the conclusion of the 3 year study was completed [27]. Quality of life progressively declined with the continuation of palliative CT and disease advancement [27]. Mayrbäurl et al. (2012) also conducted a prospective observational study at an Austrian health center that compared QOL in patients with mixed advanced stage cancer diagnoses preparing for palliative CT to healthy age and sex-matched controls. Quality of life of patients was assessed at baseline and during the palliative CT [28] with similar comparative lower QOL identified.

Groene and colleagues conducted a prospective population-based observational study of factors associated with completion of palliative CT in patients with advanced esophageal and gastric cancers in England. There were 2313 (about 24% of total) patients who received palliative CT [23]. Where data were available, only 50–60% completed treatment among those with good performance status [16]. Findings conclude low likelihood of treatment completion (53% overall).

Wintner et al. (2013) evaluated QOL in patients with advanced lung cancer who were receiving varying outpatient palliative CT treatment lines [29]. Findings showed that QOL remained essentially unchanged but was worse in those receiving advanced lines of CT [18].

## Discussion

Given progress in anti-cancer CT strategies for patients with advanced cancers, there is an increasing use of palliative CT at the end of life [15].

The purpose of this study was to evaluate relationships between use of palliative CT and QOL in patients with advanced cancer nearing end of life. Generally, the results of most of the selected studies indicated that palliative CT was associated with reduced QOL for patients. For example, such therapy was associated with increased necessity for cardiopulmonary resuscitation, mechanical ventilation, use of a feeding tube, and death in intensive care [3]. Further, such treatment was also associated with shortened survival and reduced quality of death [4].

Some patients with advanced cancer may prefer to receive aggressive treatment even if the therapies are associated with toxicities and side effects [30]. A study by Kao et al. found that patients who received palliative CT had shorter survival time and more frequent hospital admissions including unscheduled emergency department visits compared to those who did not receive CT at the end of life [26]. The goal of palliative CT is to maintain or improve QOL, improve length of survival and reduce symptoms in oncologic emergency conditions [1]. The usefulness of CT should always be considered by associated toxicities and side effects, given potential impact on QOL [1]. The presence of adverse effects from CT in cancer patients at the end of life was noted in the assessed studies [4, 9].

Early provision of palliative care with a multidisciplinary team has offered multiple benefits for patients with advanced cancer. For example, Temel and her colleagues compared two groups of patients who were newly diagnosed with metastatic cancer; one group that received palliative care with standard oncological care and the other group who received only standard oncology care. Findings demonstrated that the group that received integrated early palliative care had comparatively significant improvement in QOL, mood, and length of survival [12]. A complex issue is the use of palliative CT as a supportive strategy to improve length of survival, bolster QOL, assist with symptom management, and reduce disease progression given it may also result in more aggressive care at end of life [8]. Regimens and types of CT used in the palliative context vary. Further, it is often not clear that patients’ who receive these treatments have clearly addressed their end of life wishes and have advanced directives in place. Patients may die while receiving anti-cancer regimens because of death denial and/or communication barriers with their oncology team. For example, it may be more challenging to discuss stoppage of treatment as opposed to recommending further CT for patients who are nearing end of life [8] especially when patients are younger and have reasonable performance status. The mental health of patients at end of life and communication with their health providers may also impact the type of decisions patients will make in regards to sustaining treatment [31]. Without clear guidelines, the continued problems with medically futile treatments and untoward deaths in highly costly and non-preferred environments are likely to persist.

The review has limitations. These limitations include the possibility that articles that pertain to use of CT at end of life in patients with cancer were missed. For example, some studies may not include oral CT when evaluating use of CT at end of life [18]. Further, given the complex issues associated with patients who have advanced cancers, e.g., age, developmental stage, type of disease, comorbid conditions, etc., it can be challenging to determine best approaches to optimize individualized care at end of life. Cultural factors including the role of family caregivers may impact whether or not palliative CT is offered and utilized, and whether or not death occurs in a preferred environment. More research is needed that examines such issues across cultures, especially as more types of CT are made available to patients with advanced cancers. Research that includes diverse samples from global samples remains needed. Many of the current studies were conducted at major tertiary cancer centers so may not be reflective of practices in non-urban communities. Another limitation relates to the lag in sampling intervals among the published papers (2002–2012). Studies with more recent data collection could better reflect current practices.

## Conclusions

The results of most selected studies show that palliative CT is associated with a decrease in QOL for patients. However, studies have been conducted primarily in European and American countries, and patients' cultural backgrounds may affect quality of life at the end of life. More research is needed in developing countries, including the Middle East and Asia.

## Abbreviations

QOL: Quality of life
CT: Chemotherapy
ICU: Intensive care unit
ASCO: American Society of Clinical Oncology
HRQOL: Health related quality of life
QUADAS: Quality Assessment of Diagnostic Accuracy Studies
STARD: Standards for Reporting of Diagnostic Accuracy
U.S.: United States
QLQ-C30: Quality of Life Questionnaire-C30
ECOG: European Cooperative Oncology Group
